# Combined Ubisol-Q_10_ and Ashwagandha Root Extract Target Multiple Biochemical Mechanisms and Reduces Neurodegeneration in a Paraquat-Induced Rat Model of Parkinson’s Disease

**DOI:** 10.3390/antiox10040563

**Published:** 2021-04-06

**Authors:** Caleb Vegh, Darcy Wear, Iva Okaj, Rachel Huggard, Lauren Culmone, Sezen Eren, Jerome Cohen, Arun K. Rishi, Siyaram Pandey

**Affiliations:** 1 Department of Chemistry and Biochemistry, University of Windsor, Windsor, ON N9B3P4, Canada; veghc@uwindsor.ca (C.V.); wear@uwindsor.ca (D.W.); okaji@uwindsor.ca (I.O.); huggard1@uwindsor.ca (R.H.); culmonel@uwindsor.ca (L.C.); 2 Department of Psychology, University of Windsor, Windsor, ON N9B3P4, Canada; erens@uwindsor.ca (S.E.); jcohen@uwindsor.ca (J.C.); 3 John D. Dingell VA Medical Center; Karmanos Cancer Institute, Wayne State University, Detroit, MI 48201, USA; rishia@karmanos.org; 4 Department of Oncology, Karmanos Cancer Institute, Wayne State University, Detroit, MI 48201, USA

**Keywords:** Parkinson’s disease, oxidative stress, autophagy, neuroinflammation, paraquat, mitochondrial dysfunction, ashwagandha, Ubisol-Q_10_

## Abstract

Parkinson’s disease (PD) is characterized by progressive neurodegeneration in the substantia nigra (SN) region resulting in loss of movement coordination. Current therapies only provide symptomatic relief, and there is no agent to halt the progression of PD. Previously, Ubisol-Q_10_, a water-soluble formulation of coenzyme-Q_10,_ and ethanolic root extract of ashwagandha (ASH) have been shown to inhibit PD pathology in rodent models when used alone. Here, we evaluated the neuroprotective efficacy of oral administration of ASH and Ubisol-Q_10_ alone and in combination in a paraquat-induced PD rat model. The combined treatment resulted in better-preserved neuron morphology compared to Ubsiol-Q_10_ or ASH alone. The combination treatment enhanced activation of pro-survival astroglia and inhibited pro-inflammatory microglia. While anti-oxidative effects were seen with both agents, Ubisol-Q_10_ activated autophagy, whereas ashwagandha showed a better anti-inflammatory response. Thus, the combined treatment caused inhibition of oxidative stress, autophagy activation, inhibition of pro-inflammatory microglia, and activation of pro-survival astroglia. Consequently, paraquat (PQ)-treated rats given the combination treatment in drinking water did not show motor impairment. Based on these interesting observations, the combined treatment containing two well-tolerated natural compounds could be a more effective strategy to halt the progression of PD.

## 1. Introduction

Parkinson’s disease (PD) is a debilitating neurodegenerative disease characterized by loss of dopaminergic (DA) neurons in the substantia nigra (SN) region of the brain. The disease initially displays loss of movement coordination (resting tremors, postural instability, bradykinesia, and rigidity), which then progresses to cognitive impairments, psychiatric irregularity, and eventually morbidity [1,2]. While there are several genetic/familial factors and environmental factors linked to PD, most PD cases are sporadic and caused by unknown factors [3,4]. It is known that several similarly structured toxins such as MPTP (a synthetic heroin by-product) and paraquat/maneb/rotenone (various herbicides and pesticides) are preferentially taken up by DA neurons. These toxins then significantly elevate reactive oxygen species (ROS) when metabolized, leading to apoptosis [5,6,7,8,9,10,11,12,13,14]. Along with oxidative stress, there are several other biochemical mechanisms involved in PD, which include mitochondrial dysfunction, inhibition of autophagy leading to the buildup of defective proteins and organelles, and neuroinflammation.

Neurons are almost entirely dependent on oxidative phosphorylation to produce energy with the central nervous system requiring 20% of the total oxygen input [15,16]. As a result of high oxygen demand and since the electron transport chain (ETC) is not a perfect system, neurons have an increased tendency to produce ROS. Consistent evidence exists that links deficits of ETC complex I to excessive ROS production in both familial and sporadic forms of PD [15,16,17,18]. Furthermore, DA neurons have been shown to have reduced levels of antioxidant enzymes, further increasing their susceptibility to ROS [16]. Increased oxidative stress can eventually lead to damage to nucleic acids, proteins, and lipids. As these three constituents comprise mitochondria, ROS damage to them can lead to mitochondria dysfunction, which can then lead to further production of ROS, creating a feedback loop of increased oxidative stress and mitochondrial dysfunction [19].

As mentioned earlier, increased oxidative stress can lead to damage and the buildup of defective proteins and organelles. Cells have mechanisms to clear these defective by-products via autophagy and proteosome degradation systems. Both of these systems are involved in the degradation of defective proteins, while organelle clearing is mainly achieved via autophagy [4]. It has been shown that these systems are compromised in PD, resulting in the buildup of defective proteins and organelles leading to neurotoxicity [20].

Increasing evidence has also demonstrated the importance of neuroinflammatory responses in PD. Indeed, activated microglia were found in the brains of PD patients post-mortem [21,22,23,24]. Microglia are involved in cell debris clearance and immune response regulation due to injury/infection. This immune response can lead to apoptosis as these microglia release pro-inflammatory cytokines and ROS. It is thought that microglia are activated in earlier stages of PD alongside DA neuron loss, leading to apoptosis due to oxidative stress and inflammation [22]. Contrary to microglia activation in PD, absence of astroglia activation/number has been implicated in PD pathology [23,25,26]. Astroglia serve a variety of functions including nutrient supplementation, structural support, and secretion of pro-survival neurotrophic factors [27,28,29]. With reduced activation/numbers of protective astroglia and increased activation of microglia in PD, DA neurons are significantly more susceptible to neuroinflammation.

Although good progress has been made in providing symptomatic relief with dopamine supplements and deep brain stimulation, there is no known available remedy to stop the progression of the disease. Recently, we have observed that Ubisol-Q_10_, a water-soluble formulation of coenzyme-Q_10_ made using polyoxyethanyl-⍺-tocopheryl sebacate (PTS) [30], displays unprecedented neuroprotective properties in various models of PD. While previous preclinical studies with normal coenzyme-Q_10_ showed therapeutic efficacy at oral doses of 1600 mg/kg/day, a clinical trial [31] that had much lower required doses failed to obtain effective results. When combined with PTS, doses of coenzyme-Q_10_ were able to be reduced significantly to 6 mg/kg/day. Ubisol-Q_10_ has been shown to target mitochondrial dysfunction and oxidative stress in in vitro and in vivo models of PD [14,32,33,34,35]. Furthermore, Ubisol-Q_10_ prevented oxidative-stress-induced premature senescence and resumed impaired autophagy in Alzheimer’s Disease (AD) fibroblasts, while also improving memory and removing beta-amyloid plaques in a transgenic mouse model of AD [36,37,38]. Although not directly pertaining to PD, AD shares the same biochemical mechanisms (oxidative stress, mitochondrial dysfunction, impaired autophagy, and neuroinflammation) [39]. While Ubisol-Q_10_ seems like a promising therapeutic, it does not target all biochemical mechanisms of PD. PD should be considered a multifactorial disease, and targeting only one or a few of the biochemical mechanisms will not be enough to stop the progression of neurodegeneration.

While Ubisol-Q_10_ is able to target oxidative stress, mitochondrial dysfunction, and impaired autophagy, there is still the issue of neuroinflammation that could still be occurring. Therefore, it would be better to combine Ubisol-Q_10_ with another well-tolerated natural product that could target neuroinflammation. Recent research interest has increased with using ashwagandha root extracts to treat neurological diseases/disorders. Ashwagandha (*Withania somnifera*) is a plant of the nightshade family that has been used in Ayurveda (a traditional Indian school of medicine) as a nerve tonic for general debility, nervous exhaustion, insomnia, and memory impairment [40]. Past studies showed that various root extracts of ashwagandha were able to target oxidative stress and neuroinflammation [40,41,42,43]. The protective compounds of ashwagandha are thought to be steroidal lactones and saponins (withanolides and sitoinosides, respectively). These key components have been shown to act as antioxidants, aid in axonal regeneration, and target certain activators of inflammation [40]. We note that studies measuring ashwagandha’s efficacy alone orally administered doses that were too high for human therapeutic development. However, ashwagandha might exercise a synergistically enhanced efficacy at a much-lowered dose when combined with a water-soluble CoQ_10_ agent that could permit further human clinical trials.

In this study, based on these exciting findings, we combined Ubisol-Q_10_ and ashwagandha extract for the first time to investigate whether these two well-tolerated natural health products are more efficacious when administered together compared to the agents alone in a paraquat (PQ)-induced rat model of Parkinson’s disease. Since these treatments target different biochemical mechanisms of PD, we hypothesize that the combined formulation of Ubisol-Q_10_ and ashwagandha extract will be more effective, compared to their separate use, in protecting the brains of PQ-treated rats.

## 2. Materials and Methods

### 2.1. Ethanolic Extraction of Ashwagandha Root and Phytochemical Assessment

Ashwagandha root powder (Premier Herbal Inc., North York, ON, Canada) was soaked/stirred in anhydrous ethanol at a ratio of 1:10 (*w*/*v*) at ~70 °C for 24 h. Following 24 h, the crude extract was filtered through a P8 paper filter, and ethanol was removed using a rotary evaporator. The solid extract was then resuspended with anhydrous ethanol to a concentration of 200 mg/mL. The final suspended extracted was analyzed for several phytochemicals including withanolides and flavonoid content. Ultra-performance liquid chromatography coupled with ultra-violet spectroscopy (UPLC-UV) was used to analyze withanolide content, and colorimetric analysis based on Dowd’s reagent was used for flavonoids. Phytochemical analyses were performed by Laboratoire PhytoChemia (Chicoutimi, QC, Canada).

### 2.2. Animal Care

**Figure 1:**
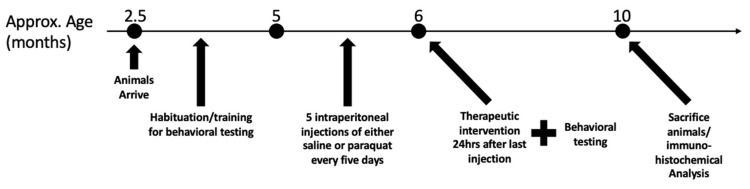
Experimental plan demonstrating the training, injection, and treatment regimen for rats.

All animal care, treatments, and procedures were approved by the University of Windsor’s Animal Care Committee in accordance with the Canadian Council for Animal Care guidelines (Animal Utilization Project Protocol #17–04). Experiments were conducted on male Long-Evans hooded rats (Charles River Laboratories, Wilmington, MA, USA). Rats arrived at 2.5 months of age and were habituated to the basic handling, feeding, transportation, and rotarod task until the age of 5 months and then underwent experimental behavioral testing following the injection procedure. Rats were housed in groups of 3–4 animals per cage for convenience and to prevent hierarchies that could arise due to the extent of neurodegeneration. Rats were individually fed outside their group cages a daily amount of 25–35 g of Purina LabDiet Rodent 5001 Chow (purchased from North American Lab Supply, Fort Worth, TX, USA) to prevent competition among animals. Animals were housed at 20 °C under a 12-hr light–dark cycle to ensure they were awake during the day for behavioral assessments. The overall schedule of the experiment, including training, injection, and treatment regimens, is summarized in Figure 1 below.

### 2.3. Injection Regimen

Rats underwent the injection regimen at 5 months of age. Rats received 5 intraperitoneal injections of PQ dissolved in 1 × phosphate-buffered saline (PBS) at 10 mg/kg body weight per injection. One injection occurred every 5 days over 20 days. Control rats received only saline injections according to the same schedule as PQ-injected rats. Animal health following injections was monitored daily by the University of Windsor’s Animal House veterinarians.

### 2.4. Drinking Water Treatment

Animals were provided the following treatments in their drinking water 24 h after the last day of injections: saline-injected rats were given plain drinking water (*n* = 7); saline-injected rats were given the tonic (combination of 50 μg/mL Ubisol-Q_10_ (provided by Next™ Remedies, Toronto, ON, Canada) and 2 mg/mL ethanolic ashwagandha extract (ASH)(*n* = 5); PQ-injected rats were given plain drinking water (*n* = 9); PQ-injected were rats given PTS carrier (*n* = 6); PQ-injected were rats given 50 μg/mL Ubisol-Q_10_ (*n* = 7); PQ-injected rats were given 2 mg/mL ASH (*n* = 8); PQ-injected rats were given the tonic (*n* = 8). Groups in which ASH was not provided had 1% anhydrous ethanol added to the drinking water to account for 1% ethanol present in the water when ASH was added. Fresh drinking solutions were provided every 3–4 days. Treatment continued for 4 months during which behavioral assessments were conducted. Following 4 months, animals were sacrificed, and their brains were extracted for biochemical analysis.

### 2.5. Behavioral Assessments

**Figure 2:**
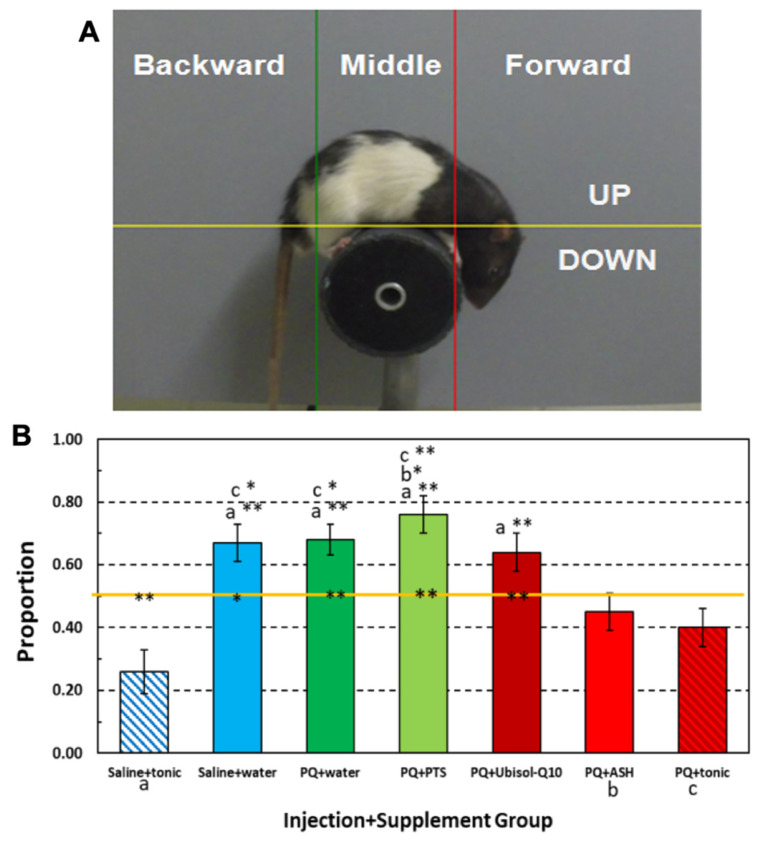
(**A**) JPEG image from a digital 2-min video of a rat on a thin rotarod with vertical and horizontal guideline lines for determining body orientation (forward, backward) and head position based on nose location (see text for further details). (**B**) Mean proportion of time rats in each group spent walking in a head-down position on a 6-rpm rotating rod as determined by nose location above and below the horizontal orange line. Three groups marked along the X-axis are: a = Saline+tonic (one of two saline-injected control groups), b-PQ+ASH (the second of three neuro-protected treatment groups), and c- PQ+ tonic (the third of three neuro-protected treatment groups). Only these three groups were so marked because each of them spent significantly less time walking in the head-down position than any of the other four unmarked groups as indicated by its mark on top of any of four unmarked groups’ mean proportion bars. Vertical error lines = ±1 SE (standard error of the mean). * *p* < 0.05; ** *p* < 0.01. The *p* values that appear at the top of any of the four unmarked group’s bar assess which a, b, or c group significantly differs that unmarked groups mean value as determined from supplementary pair-wise comparisons between groups. Those *p* values occurring on the horizontal line within or near each group’s mean data bar indicates which group’s rats spend significantly more or less time in a head-down walking position.

Motor/balance coordination was measured on the rotarod. The rats’ ability to maintain balance on a slowly rotating cylinder was measured with a rotarod apparatus similar to that previously described in [44]. Our modified rotarod apparatus is shown in Figure 2 It consisted of either a 9-cm diam (wide) or a 5-cm diam (thin), 15-cm long black polyethylene dowel with metal strips embedded lengthwise in its surface to prevent rats from slipping off the rotating rod. Either rod could be attached to a variable speed motor hidden behind a vertical 30 × 48 cm flat grey plastic panel. During the course of the experiment, we ran different 2-min sessions with either rod that rotated counterclockwise at 6, 9, or 12 rpm during a session. A digital video camera was positioned 1 m in front of and in level with the rod. Regular fluorescent ceiling lighting and a 60-W lamp approximately 3 m in front of the apparatus illuminated it. The MPEG recordings of each rat’s rotarod performance were converted to JPEG images at a rate of 5 frames per second. We analyzed each animal’s movements over its last 600 frames (120 s). The position of the tip of the nose was tracked on a 450 × 450-pixel Cartesian system of coordinates with tracking software (Seven Software, Inc., Rockaway, NJ, USA). The grid was divided by a horizontal line above the rotarod and by two vertical lines, one to the right and the other to the left. The proportion of frames in which the animal’s nose is beyond the right and beyond the left of these vertical lines was our measure of the proportion of time the animal spent walking forward and backward, respectively. We note from past experience that the time the animal’s nose is between the two vertical lines, as it is when turning around, does not account for more than 10% of its total time on the rotarod. Statistical Product and Service Solutions (SPSS; IBM, Armonk, NY, USA) was used to calculate statistical significance using one-way ANOVA.

### 2.6. Tissue Preparation for Immunohistochemistry

Following the experimental period, rats were euthanized while under anaesthetization via 3% isoflurane at a flow of 2 L oxygen/min. Once the animal showed a lack of withdrawal reflex (indicating stage 3 anesthesia/lack of pain), the entire animal body was perfused with ice-cold PBS containing 28 ug/mL heparin (Sigma-Aldrich, Oakville, ON, Canada, Cat. No. H3393), followed by fixation with ice-cold 10% formaldehyde made in PBS. Following perfusion, brains were dissected and stored in 10% formalin at 4 °C. To prepare for sectioning, brains were incubated in 30% sucrose (*w*/*v* in PBS) until brains sank in the solution. Following sucrose incubation, brains were cryosectioned at 30 μm thickness with Shandon^TM^ M-1 embedding matrix (Thermo Scientific, Mississauga, ON, Canada, Cat. No. 1310 TS) onto glass microscope slides.

### 2.7. Immunohistochemistry (Colorimetric)

Sections were washed for 5 min twice with tris-buffered saline (TBS), followed by incubation with 0.3% H2 O2 to block endogenous peroxidase activity. Sections were rinsed for 5 min twice with TBS, followed by a 30-min block with Dako serum-free protein block (Agilent Technologies Canada Inc., Mississauga, ON, Canada, Cat. No. X0909) and normal serum according to instructions of the Vector Laboratories VECTASTAIN Elite ABC-HRP kit, Peroxidase (rabbit IgG; MJS BioLynx Inc., Brockville, ON, Canada, Cat. No. VECTPK4001). Tissue sections were incubated overnight at 4 °C, with tyrosine hydroxylase (TH) primary antibody (rabbit IgG; 1:1000; Cat. No. P40101–150) (Pel-Freez Biologicals, Rogers, AR, USA). Tissue sections were washed for 5 min twice with TBS, followed by incubation with secondary biotinylated antibody according to instructions from the VECTASTAIN Elite ABC-HRP Peroxidase kit. Sections were washed twice with TBS for 5 min, then incubated with avidin-conjugated horseradish peroxidase from the VECTASTAIN Elite ABC-HRP Peroxidase kit for 45 min. Sections were washed twice with TBS for 5 min and incubated with 3,3′-diaminobenzidine (DAB) stain solution according to the Vector Laboratories DAB Peroxidase Substrate kit (MJS BioLynx Inc., Brockville, ON, Canada, Cat. No. SK-4100). Sections were dehydrated with two 5-min washes in anhydrous ethanol then a 7-min xylenes wash, followed by coverslipping using Permount^®^ mounting medium (Fisher Scientific Canada, Ottawa, ON, Canada, Cat. No. SP15-500). Cells were imaged using bright-field microscopy via a Leica DMI6000 B inverted microscope (Leica Microsystems, Concord, ON, Canada).

### 2.8. Immunohistochemistry (Fluorescent)

Sections were washed for 5 min twice with TBS, followed by incubation with Dako serum-free protein block (Agilent Technologies Canada Inc., Mississauga, ON, Canada, Cat. No. X0909). Tissue sections were then incubated overnight at 4 °C in the following primary antibodies: glial fibrillary acidic protein (GFAP) (rabbit IgG, 1:500; Novus Biologicals, Centennial, CO, USA, Cat. No. NB300-141), ionized calcium-binding adapter molecule 1 (Iba-1) (rabbit IgG, 1:300; Novus Biologicals, Cat. No. NB100-1028), tyrosine hydroxylase (rabbit IgG, 1:1000; Pel-Freeze Biologicals, Cat. No. P40101-150), beclin-1 (mouse IgG, 1:500; Santa Cruz Biotechnology, Dallas, TX, USA, Cat. No. sc-48342), pro-brain-derived neurotrophic factor (pro-BDNF) (mouse IgG, 1:500; Santa Cruz Biotechnology, Cat. No. sc-65513), glial-derived neurotrophic factor (GDNF) (mouse IgG, 1:500; Santa Cruz Biotechnology, Cat. No. sc-13147), 4-hydroxynonenal (4-HNE) (rabbit IgG, 1:500; Abcam Inc., Cambridge, UK, Cat. No. ab46545), and cell division cycle and apoptosis regulator 1 (CARP1) (rabbit IgG, 1:1000; provided by Dr. Arun Rishi of Wayne State University). The following day, tissue sections were washed for 5 min twice with TBS and incubated at room temperature for 2 h in the following secondary antibodies: Vector Laboratories fluorescein horse anti-mouse IgG (1:500; MJS BioLynx Inc., Brockville, ON, Canada, Cat. No. FI-2000) and Alexa Fluor^TM^ 568 goat anti-rabbit IgG (Thermo Scientific Canada, Brockville, ON, Canada, Cat. No. A11011). Sections were then washed twice for 5 min in TBS followed by coverslipping with VECTASHIELD^®^ Vibrance^®^ antifade mounting medium with DAPI (4′,6-diamidino-2-phenylindole) (MJS BioLynx Inc., Brockville, ON, Canada, Cat. No. VECTH18002). Tissue sections were imaged using epifluorescence microscopy via a Leica DMI6000 B inverted microscope (Leica Microsystems, Concord, ON, Canada). Fluorescence was quantified in images captured using ImageJ software. For each group, the fluorescence was quantified for each specified protein per overall SN image captured. Fluorescent analyses were performed on 3 sections per animal.

## 3. Results

### 3.1. Phytochemical Content of Ashwagandha Extract

Following extraction of the ashwagandha root powder, we sought to determine the concentration of various withanolides and a flavonoid of the extract. Phytochemical analysis was performed by Laboratoire PhytoChemia. A summary of the phytochemical analysis of the extract is provided below in Table 1. Withanolide content was determined via UPLC-UV. Withaferin A concentration was 13.6 mg/mL, 12-Deoxy-withastramonolide was 3.8 mg/mL, withanolide A was 5.5 mg/mL, and withanolide B was 1.9 mg/mL. Combined together, total withanolides account for approximately 12.4% of the extract composition (24.8 mg/mL of total withanolides in the 200 mg/mL extract). Flavonoid content was determined via colorimetric analysis based on Dowd’s reagent. Quercetin concentration was determined to be 1.63 mg/mL per 100 mL of extract.

### 3.2. Effect of Ubisol-Q_10_ and Ashwagandha Combined Compared to Agents Alone in Protecting DA Neurons from PQ-Induced Neurotoxicity

**Figure 3:**
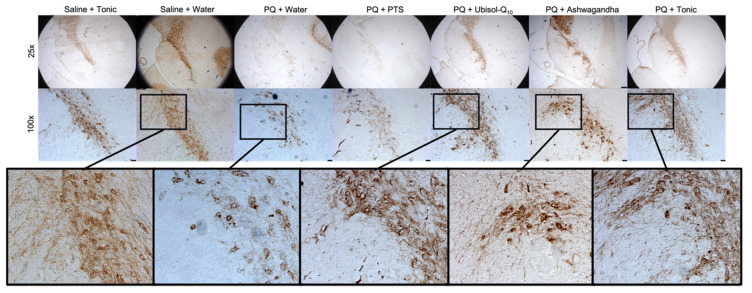
Effect of Ubisol-Q_10_ and ashwagandha on neurodegeneration of substantia nigra neurons in saline/paraquat (PQ)-injected rats. Light micrographs of midbrain sections showing tyrosine hydroxylase (TH) positive neurons at 25× (scale bar = 250 microns) and 100× (scale bar = 50 microns) magnification in the substantia nigra (SN).

**Figure 4:**
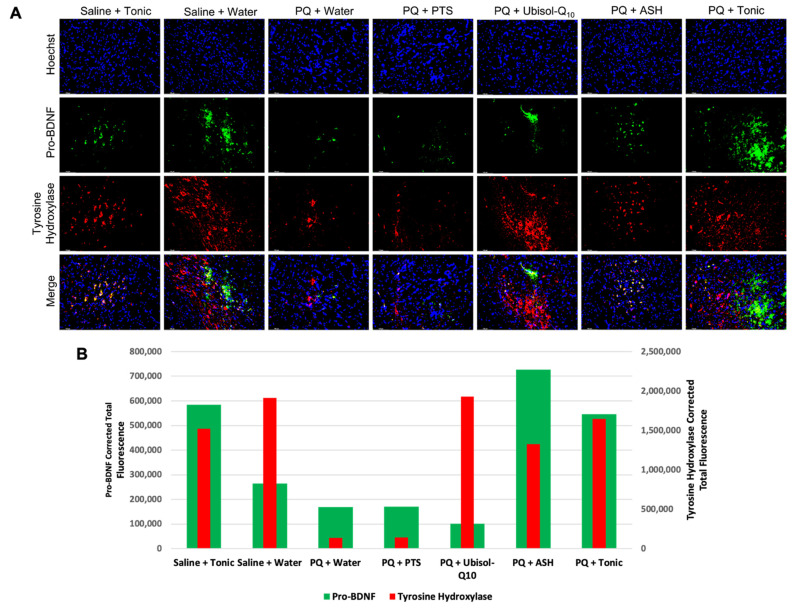
Effect of Ubisol-Q_10_ and ashwagandha on dopaminergic (DA) neuron apoptosis and pro-brain-derived neurotrophic factor (pro-BDNF) secretion in saline/PQ-injected rats. (**A**) Immunofluorescent staining at the tip of SN in midbrain sections probing for pro-survival neurotrophic factor, pro-BDNF, and tyrosine hydroxylase (TH) and (**B**) Quantification of fluorescent of pro-BDNF and TH. Nuclei were counterstained with DAPI. Micrographs were taken at 100× magnification. Scale bar = 100 microns.

Here, we combined Ubisol-Q_10_ and ASH for the first time to examine if the agents combined are more effective compared to their individual use. We observed that rats exposed to PQ and given only plain drinking water or the PTS vehicle had significant neurodegeneration in the SN, as indicated by reduced immunoreactivity for tyrosine hydroxylase (TH) (a marker of DA neurons [34]) (Figure 3). Rats exposed to PQ and given either Ubisol-Q_10_ or ASH alone had significant protection for their DA neurons (Figure 3). Interestingly, while ASH protected fewer AD neurons than Ubisol-Q_10_ (reduced tyrosine hydroxylase immunoreactivity), it appeared to better maintain the morphology of protected neuron as shown by apparently greater abundance of fibers extending from the cell bodies, appearing more similar to neurons in the animals injected with only saline and given plain drinking water (Figure 3). When PQ-treated rats were given the tonic treatment (combination of Ubisol-Q_10_ and ASH), we saw the benefits of both treatments in that Ubisol-Q_10_ maintained the number of DA neurons while ASH maintained their morphology. Indeed, the PQ + tonic rats’ SNs appeared almost identical to the control (saline + water) group (Figure 3). Furthermore, we also wanted to confirm that the tonic treatment of Ubisol-Q_10_ and ashwagandha was not toxic to healthy animal brains. Indeed, the tonic treatment did not result in any observable neurodegeneration of the SN in animals injected with saline (Figure 3). Along with colorimetric immunohistochemistry to detect TH, we also performed immunofluorescent staining (Figure 4). While immunofluorescent images were taken towards the tip of the SN instead of towards the overall/center of the SN, as in Figure 3, similar observations were made in the rats’ brains. PQ-treated animals given plain water or PTS exhibited reduced levels of fluorescence for TH compared to the two saline-injected groups. Treatment with Ubisol-Q_10_, ASH, or the tonic had higher amounts of TH fluorescence compared to PQ animals fed plain water or PTS.

### 3.3. Ubisol-Q_10_ and ASH Antioxidant Effects against PQ-Induced Neurotoxicity

**Figure 5:**
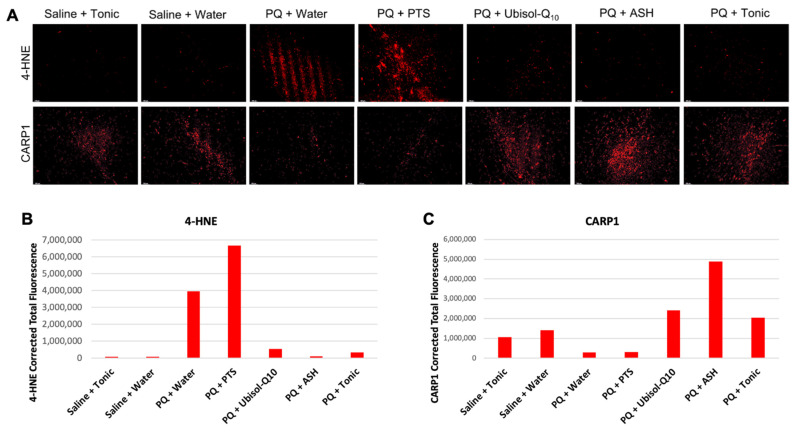
Effect of Ubisol-Q_10_ and ashwagandha on oxidative stress and apoptosis regulation. (**A**) Immunofluorescent staining of the SN in midbrain sections probing for oxidative stress marker and lipid peroxidation by-product 4-hydroxynonenal (4-HNE) and cell division cycle and apoptosis regulator 1 (CARP1). (**B**,**C**) Fluorescent quantification of 4-HNE and CARP1, respectively. Micrographs were taken at 100× magnification. Scale bar = 100 microns.

It is well known that exposure to PQ results in increased production of ROS [10,11,14]. We observed increases in levels of the lipid peroxidation product 4-hydroxynonenal (4-HNE) in the brains of PQ-treated rats given plain or PTS-supplemented drinking water (Figure 5A,B). When these animals were given Ubisol-Q_10_, ASH, or the tonic, 4-HNE levels were reduced, appearing similar to the saline-injected rats given plain water or the tonic as indicated by reduced immunoreactivity for 4-HNE (Figure 5A,B).

### 3.4. CARP1 Expression in Response to PQ Insult and Treatment with Ubisol-Q_10_ and Ashwagandha Extract

Cell division cycle and apoptosis regulator 1 (CAPR1) is involved with regulating cell death and is known to be a positive regulator of apoptosis [45]. Here, we wanted to observe the status of CARP1 and determine its role in PQ-mediated neurotoxicity. Interestingly, CARP1 levels were reduced in the SN of PQ-injected rats fed plain water or PTS compared to the saline groups (Figure 5A–C). Furthermore, PQ-injected rats fed Ubisol-Q_10_, ASH, or the tonic had higher levels of CARP1 compared to the saline groups (Figure 5A–C).

### 3.5. Effect of Ubisol-Q_10_ and Ashwagandha Treatment on Beclin-1 Expression

**Figure 6:**
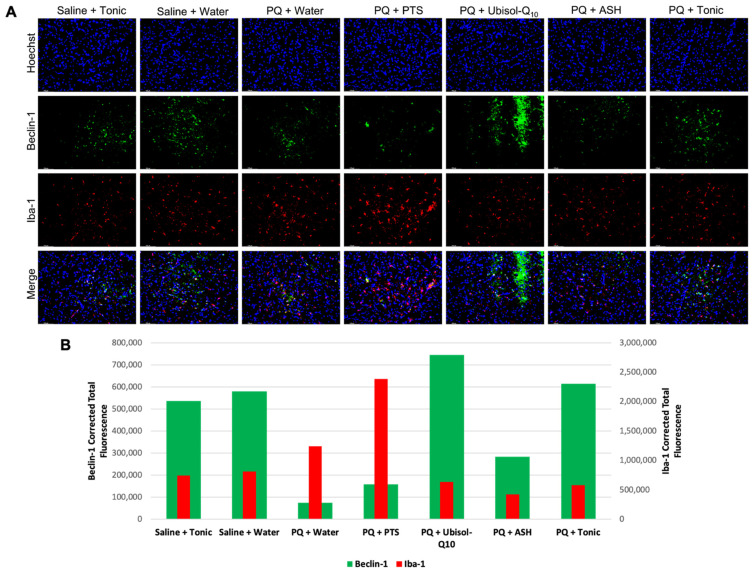
Effect of Ubisol-Q_10_ and ashwagandha on activation of pro-inflammatory microglia and autophagy. (**A**) Immunofluorescent staining of the SN in midbrain sections probing for Beclin 1, a major regulator of autophagy, and ionized calcium-binding adapter molecule 1(Iba-1), a marker of microglia activation. (**B**) quantification of fluorescence of Beclin 1 and Iba-1. Nuclei were counterstained with DAPI. Micrographs were taken at 100× magnification. Scale bar = 100 microns.

We have shown that when AD fibroblast and transgenic AD mice were treated with Ubisol-Q_10_, the major autophagy regulator Beclin 1 was upregulated compared to untreated groups [36,37,38]. As mentioned earlier, PD and AD share several biochemical mechanisms leading to neurodegeneration including impaired autophagy. We wanted to investigate whether this same mechanism was activated in the rats of this study. Indeed, we observed that animals injected with PQ and fed plain or PTS-supplemented water had decreased levels of Beclin 1 compared to saline-injected animals (Figure 6). We saw Beclin 1 expression was increased in rats given Ubisol-Q_10_ and to a lesser extent with the tonic (Figure 6). While not as effective as Ubisol-Q_10_, PQ-injected rats fed ASH also had elevated levels of Beclin 1 compared to PQ rats fed plain water or PTS (Figure 6).

### 3.6. Inflammatory Status in the Brains of Rats in Response to PQ Neurotoxicity and Treatment with Ubisol-Q_10_ and Ashwagandha Extract

**Figure 7:**
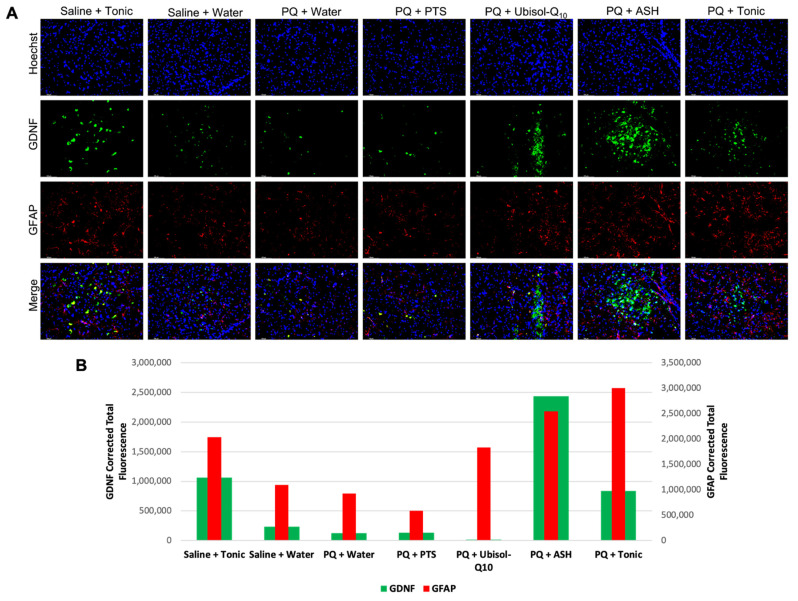
Effect of Ubisol-Q_10_ and ashwagandha extract on activation of pro-survival astroglia and secretion of glial-derived neurotrophic factor. (**A**) Immunofluorescent staining of the SN in midbrain sections probing for pro-survival neurotrophic factor glial-derived neurotrophic factor (GDNF) and glial fibrillary acidic protein (GFAP), a marker of astroglia activation. (**B**) Quantification of fluorescence of GDNF and GFAP. Nuclei were counterstained with DAPI. Micrographs were taken at 100 × magnification. Scale bar = 100 microns.

We examined the status of both microglia and astroglia in response to treatment with Ubisol-Q_10_ and ashwagandha (Figure 6 and Figure 7). We saw that in rats injected with PQ and only fed plain water or PTS, levels of pro-inflammatory microglia were elevated compared to saline-injected animals as indicated by elevated immunoreactivity for Iba-1 (Figure 6). We saw that presence of active pro-survival astroglia was reduced in PQ-treated rats fed plain water or PTS, as indicated by measured levels of GFAP. With either treatment of Ubisol-Q_10_, ASH, or the tonic, microglia activation was reduced compared to PQ-treated rats given plain water or PTS. Furthermore, microglial activation was even reduced slightly in saline-injected animals given the tonic compared to the saline animals given water. Compared to microglia (Figure 6), an opposite outcome was observed with astroglia activation, as shown in Figure 7. Animals injected with PQ and fed plain water or PTS had reduced activation of astroglia compared to saline-injected animals. PQ-injected animals given Ubisol-Q_10_ or ASH showed significant increases in activation of astroglia compared to the PQ animals fed plain water or PTS. Furthermore, when combined, Ubisol-Q_10_ and ASH had an even greater effect on astroglial activation compared to the agents alone (Figure 7). Interestingly, we observed that the saline-injected animals given the tonic had around double the amount of astroglia activation compared to saline animals given plain water.

### 3.7. Modulation of Levels of Neurotrophic Factors, GDNF and Pro-BDNF in Response to PQ Insult and Treatment with Ubisol-Q_10_ and Ashwagandha Extract

Along with looking at the status of astrocytes, we also probed for both GDNF and pro-BDNF to examine whether neurotrophic factor levels are affected by astrocyte activity. Interestingly, all animal groups that had ASH in their drinking water exhibited elevated levels of GDNF compared to all other groups (Figure 7). Similarly, pro-BDNF was also elevated in all groups with drinking water containing ASH compared to groups without ASH (Figure 4).

### 3.8. Effect of Ubisol-Q_10_ and Ashwagandha Extract on PQ-Induced Motor Deficits

Chronic PQ or MPTP exposure in rats or mice is known to cause impaired motor performance on the rotarod task [14,35,36,46]. As already discussed, we focused our analysis on comparing the groups’ proportion of head-down positions as they walked on a slowly rotating (6 rpm), 5-cm cylinder (rotarod) (Figure 2). A one-way ANOVA for groups uncovered a significant effect for this factor, *F*_6, 43_ = 8.29, *p* = < 0.001, ή_p_^2^ = 0.54, observed Power = 1. Post-hoc pair-wise comparisons between groups revealed that animals given a tonic supplement after receiving either saline or PQ injections (a- or c-labeled groups) spent significantly less time walking with their heads in a downward position than rats given only water or PTS after receiving PQ injections and, unexpectedly, than rats that continued to receive only water after being injected with saline or that received Ubisol-Q_10_ after being injected with PQ. Although PQ-injected rats given ashwagandha (b-labeled group) spent significantly less time walking on the rotarod in a head-down position than the solely PQ-injected rats given PTS, they, along with PQ or saline-injected rats given the tonic, did not significantly exceed more than 50% of their time doing so, compared to PQ-injected rats given PTS, water, or Ubisol-Q_10_, or compared to saline-injected rats given water, who spent significantly more than 50% of their time in a head-down position. Indeed, only saline-injected rats given the tonic solution spent significantly less than 50% of the time in a head-down position. We must point out that we had two groups of healthy saline-injected animals (i.e., tonic fed or plain water). We were not expecting any motor deficits in the saline-injected animals, and this is true for the saline-injected, tonic-fed animals. However, saline-injected, water-fed animals did show some deficits, which was unexpected.

## 4. Discussion

In this study, we found that Ubisol-Q_10_ and ethanolic ashwagandha extract (ASH) combined, two simple and well-tolerated nutraceuticals, contributed in a complementary way to target the multiple biochemical mechanisms implicated in PD. With PD being a multifactorial disease, the two agents combined were more effective at halting the progressive neurodegeneration of PD compared to the agents administered alone. Furthermore, by combining the agents, each targeting different mechanisms of PD, we were able to use a lower dose of one known neuroprotectant, ASH, compared to those in other studies that used ASH alone.

Paraquat is an herbicide and environmental toxin well known to cause in people exposed to it the development of Parkinson’s disease [10,11,12,13]. Other mammals such as rats are also known to develop similar neurodegeneration in the SN when exposed to PQ. The toxic effects of PQ have been studied in rats, and a well-established model has been developed [34]. After chronic exposure to low doses of PQ, progressive neurodegeneration of DA neurons occurs in the brains of rats. While Ubisol-Q_10_ did target some mechanisms of PD, it did not target all. With PD being a multifactorial disease, targeting only one or a few mechanisms is not enough to halt neurodegeneration. We sought to combine Ubisol-Q_10_ with another reagent, one that may target the other mechanisms of PD that Ubisol-Q_10_ did not as effectively such as inflammation. As a result, we combined Ubisol-Q_10_ with ethanolic extract of ashwagandha, and, indeed, we found that DA neurons were better protected by the tonic compared to the agents alone (Figure 3). While Ubisol-Q_10_ was able to maintain the overall amount of DA neurons in the substantia nigra of PQ-treated rats, their morphology was damaged compared to saline-injected animals, as there were very few nerve fibers extending from their cell bodies. ASH, while not as effective at maintaining the same amount of DA neurons, better protected the overall morphology of the neurons as there were more fibers that extended off the cell bodies similar to that in the saline-injected animals. When combined, the benefits of Ubisol-Q_10_ and ASH were seen in the brains of PQ-treated rats (neuron numbers maintained by Ubisol-Q_10_ and cell morphology protected by ASH) (Figure 3). Furthermore, we found that the tonic of Ubisol-Q_10_ and ASH had no ill effects on DA neurons in saline-injected rats.

4-HNE, a lipid peroxidation by-product and oxidative stress marker was almost completely eliminated in the SN of PQ-treated rats fed Ubisol-Q_10_ (Figure 5). Similarly, ASH also reduced levels of 4-HNE, as seen in Figure 5, confirming the previously reported antioxidant effects of ashwagandha extracts [41]. Furthermore, Ubisol-Q_10_-fed animals (either given saline or PQ injections) showed elevated levels of Beclin 1, a major autophagy regulator, indicating increased levels of autophagy (Figure 6). These are very exciting results, as upregulation of Beclin 1 by Ubisol-Q_10_ was only previously reported in AD fibroblasts and transgenic AD mice [38]. Thus, our results further confirm the autophagy activating mechanism of Ubisol-Q_10_ via upregulation of Beclin 1. Activation of autophagy is especially important in PD as defective mitochondria are known to accumulate, which can result in further production of ROS leading to apoptosis [15,19,20].

In this study, we sought to determine the status of microglia and astroglia in response to PQ insult and Ubisol-Q_10_ and ASH treatment. We observed enhanced microglial activation in PQ-treated rats fed plain water or PTS compared to saline-injected animals (Figure 6). Treatment with either Ubisol-Q_10_, ASH, or the tonic resulted in drastic inhibition of active microglia. Furthermore, microglial activation levels were similar to levels of 4-HNE. It is possible that microglia could be involved, along with PQ, in inducing oxidative stress. Along with microglia, we also investigated the status of astroglia. Opposite to our observations of microglia, levels of active astroglia were reduced in PQ-treated rats fed plain water or PTS compared to rats injected with saline (Figure 7). We saw an increase in astroglia activation in both Ubisol-Q_10_ and more so in ASH (Figure 7). When combined together, the tonic showed increased astrogliosis compared to the agents alone. Saline-injected animals given the tonic had elevated levels of astroglia compared to the saline-injected animals fed plain water. Astroglia are also known to secrete several pro-survival neurotrophic factors including GDNF and BDNF [27,28,29]. While both Ubisol-Q_10_ and ASH resulted in increased astrogliosis, only groups where ASH was given showed increased levels of GDNF and pro-BDNF (precursor to BDNF, which eventually gets cleaved to form BDNF) (Figure 4 and Figure 7). This could indicate that ASH not only acts as an anti-inflammatory by inhibiting pro-inflammatory microglia and activating pro-survival astroglia but also stimulates another pro-survival response.

Previously, CARP1 was considered to be mainly involved in mediating apoptosis [45]. Here, we observed that CARP1 was down-regulated in PQ-treated rats given plain water or PTS compared to all other groups (Figure 5A–C), suggesting that CARP1 is acting as a pro-survival regulator in our model of PD. While surprising, this observation is not totally unexpected. CARP1 has been shown to be involved in NR3A (NMDA-type glutamate receptor subunit) synaptic signaling, along with regulating β-catenin in colon cancer metastasis, co-activating GR (glucocorticoid receptor) signaling during adipogenesis, or neurogenin3-mediated pancreatic endocrine differentiation [47,48,49,50]. CARP1 also interacts with Necdin to regulate myoblast survival [51]. Furthermore, CARP1 interaction with NEMO (NF-kappa-B essential modulator) is involved in the regulation of pro-inflammatory NF-_K_B survival signaling [45]. CARP1-NEMO signaling is proposed to be involved in the regulation of DNA-damage-induced survival signaling. CARP1 is also shown to be a co-activator of the cell cycle regulatory APC/C E3 ligase [52]. APC/C E3 ligase is a critical regulator of G2/M transition, where APC/CCDC20 E3 ligase regulates cyclin B degradation to manage G2 exit. It could be possible that elevated levels of CARP1 in our study helped sustain optimal APC/C CDC20 activity for enhanced G2/M exit and cell cycle progression and survival signaling.

One of the most striking and commonly observed symptoms in humans with PD is their tendency to form a forward lean in their gait [53]. A similar head-down symptom was first observed in PQ-injected rats on the rotarod [46]. Although in a later study from our laboratory that employed a more standardized rotarod test, this symptom was not replicated in PQ-exposed rats, but rather the animals not given any neuroprotection from Ubisol-Q_10_ displayed less time walking backward than their neuroprotected counterparts or saline-injected control rats [14]. Despite the fact that the present study did not replicate these body orientation effects; it did reveal head-down position differences as a function of rats’ injection/water supplement condition, as we already described. It seems that the tonic composed of Ubisol-Q_10_ and ashwagandha was more potent in limiting PQ-injected rats’ time spent walking in the head-down position than when ashwagandha or Ubisol-Q_10_ were administered separately. Of these two separately administered neuro-protectants, only ashwagandha produced any statistically significant head-down limiting effects. Thus Ubisol-Q_10_ alone may be a less effective behavioral treatment for PD if not combined with a greater anti-inflammatory agent. The question remains whether such an anti-inflammatory agent must also have unrelated neurotrophic properties to provide a more potent treatment with another non-anti-inflammatory neuroprotectant such as Ubisol-Q_10_. The saline-injected rats (non-lesioned) given the tonic or water should not show any deficits in motor balance. Indeed, the saline-injected, tonic-fed rats showed better motor balance performance than PQ-injected rats. However, the saline-injected rats given only water showed deficiency in motor balance, which was an unexpected anomaly and difficult to explain. This raises the question of whether either of the tonic’s components given separately offer equally potent effects in rats with unaffected brains.

Following extraction, the ashwagandha extract was subjected to phytochemical analysis. It was determined that the total extract comprised 12.4% withanolides. The extract also was determined to contain 1.63 mg/mL quercetin equivalents per 100 mL of the total extract. Both flavonoids and withanolides are known to have potent anti-inflammatory properties [40,54], and we reported similar observations with our extract, as mentioned above. Previous studies used ashwagandha extract concentrations that are too high for human consumption/clinical development [40,41,42,43]. In this study, we were able to use significantly lower doses of ethanolic ashwagandha extract that still had neuroprotective effects against PQ toxicity. While the extract was not as effective by itself in protecting DA neurons, the extract combined with Ubisol-Q_10_ was more effective at protecting DA neurons compared to either agent alone. Additionally, while our extract did not have as high a withanolide content compared to other groups [43], our extract did not require the use of toxic solvents, and by combining the extract with Ubisol-Q_10_, the dose of the extract was able to be drastically reduced to a concentration (2 mg/mL) suitable for human clinical development.

## 5. Conclusions

In this study, we combined Ubisol-Q_10_ and ashwagandha root extract for the first time as a potential therapeutic for PD so as to determine whether the agents combined are more effective compared to the agents alone. We found the tonic of Ubisol-Q_10_ with ethanolic extract of ashwagandha root extract combined was more efficacious in protecting DA neurons in a PQ-induced rat model of PD compared to the therapeutic agents alone. Ubisol-Q_10_, ashwagandha, or the tonic were able to reduce oxidative stress as well as stimulate the activation of the apoptosis regulator CARP1 in the brains of the rats. Furthermore, we found that animals given Ubisol-Q_10_ or the tonic showed increased expression of the autophagy regulator Beclin 1. This is the first time Ubisol-Q_10_ was shown to enhance autophagy activation in the brains of PQ rat models of PD. Activation of pro-survival astroglia and inhibition of pro-inflammatory microglia was observed in the brains of PQ-exposed rats given ashwagandha or the tonic, which also coincided with the expression of pro-survival neurotrophic factors GDNF and pro-BDNF only in the brains of rats given ashwagandha or the tonic. Thus, the tonic of Ubisol-Q_10_ and ashwagandha was more efficacious at protecting PQ-treated rats from the biochemical mechanisms of PD compared to the agents alone. The reason for the better neuroprotective results with the tonic is likely due to the fact that the two agents are targeting different biochemical etiologies of PD. With both Ubisol-Q_10_ and ASH being simple nutraceutical compounds and GRAS (generally regarded as safe)-approved, they can also be taken over long periods of time without serious side effects. Thus, Ubisol-Q_10_ and ashwagandha root extract could prove to be a promising therapy for PD that could halt neurodegeneration and improve quality of life.

## 6. Patents

Ubisol-Q_10_ is a patented formulation.

## Figures and Tables

**Table 1:** Withanolide and flavonoid content of ethanolic ashwagandha extract.

Withanolides					Flavonoids
Content (mg/mL)					Content (mg/100 mL)
	Withaferin A	12-Deoxy-withastramonolide	Withanolide A	Withanolide B	Quercetin
Assay 1	13.0	3.7	5.3	1.8	Mean 1.63 ± 0.07
Assay 2	13.9	4.0	5.6	2.0	
Assay 3	13.8	3.8	5.6	1.7	
Assay 4	13.6	3.9	5.5	1.8	
Mean ± standard deviation	13.6 ± 0.4	3.8 ± 0.1	5.5 ± 0.1	1.9 ± 0.1	

## Data Availability

The data from this work are available.
